# The Correlation between Time in Range and Diabetic Microvascular Complications Utilizing Information Management Platform

**DOI:** 10.1155/2020/8879085

**Published:** 2020-12-15

**Authors:** Xia Sheng, Guo-Hui Xiong, Peng-Fei Yu, Jian-Ping Liu

**Affiliations:** ^1^Department of Endocrinology, Third Affiliated Hospital, Nanchang University, Nanchang 330008, China; ^2^Nanchang Hongdu Hospital of Traditional Chinese Medicine, Nanchang 330000, China; ^3^Department of Endocrinology, Second Affiliated Hospital, Nanchang University, Nanchang 330008, China

## Abstract

**Background:**

In recent years, the time of blood glucose within the target range is a new research hotspot in blood glucose management. TIR is expected to be a novel indicator for evaluating the efficacy of glycemic control and predicting diabetic complications. However, its relationship with diabetic complications has not been fully elucidated.

**Objective:**

To explore the relationship between time in range (TIR) and glycosylated hemoglobin (HbA1C) through the information big data management platform. Possible association between TIR and diabetic microvascular complications (retinopathy, nephropathy, and neuropathy) was investigated, attempting to provide theoretical basis for the clinical application of TIR and to explore the TIR control scope suitable for diabetic patients.

**Methods:**

A total of 5,644 type 2 diabetic patients hospitalized in the Department of Endocrinology, the Second Affiliated Hospital of Nanchang University, were selected from April 2017 to June 2020. Fingertip capillary blood glucose monitoring (FCGM) was monitored for a total of 455,664 times, and patients who are nondiabetic, pregnant, or with diabetic ketosis were excluded. Patients with 7 blood glucose points monitored for at least three consecutive days were selected as subjects in the study. 1,895 males and 1,513 females with diabetes were included, with an average age of (59.74 ± 13.40) years old and an average course of disease of 8.28 ± 7.11 years. The proportion of time in range (TIR) (70∼180 mg/dl) within the target range and the correlation between TIR and HbA1C were analyzed, as well as the relationship between TIR and the risk of diabetic complications.

**Results:**

(1) The average of TIR and HbA1C was 49.65 ± 23.36% and 8.92 ± 2.49%, respectively, and was linearly correlated. With the decrease of TIR, HbA1C increased significantly, and the difference was statistically significant (*P* < 0.01, *R*^2^ = 0.458). The correlation coefficient of mean TIR with mean HbA1C was −0.626. (2) There were 836 patients diagnosed with diabetic nephropathy (DN). The difference of TIR value between DN and non-DN was significant (*T* = 2.250, *P* < 0.05). Risk assessment showed the lower the TIR was, the higher the risk of DN was. TIR less than 40% was a risk factor for DN (OR = 1.249, 95% CI: 0.915–1.375). (3) There were 1,296 patients diagnosed with diabetic peripheral neuropathy (DPN). The difference of TIR value between DPN and non-DPN was significant (*T* = 3.844, *P* < 0.01). TIR value less than 70% was a risk factor for DPN (OR = 1.030, 95% CI: 0.769–1.379). (4) There were 2,077 patients diagnosed with diabetic retinopathy (DR). The difference of TIR value between DPN and non-DPN was significant (*T* = 3.608, *P* < 0.01). TIR value less than 50% was a risk factor for DR (OR = 1.092, 95% CI: 0.898–1.264). *Summary*. TIR may serve as a reference index for short-term blood glucose control, strongly reflecting the clinical blood glucose regulation and predicting the risk of diabetic microvascular complications.

## 1. Introduction

With the increasing use of continuous blood glucose monitoring (CGM) in recent studies, the time of target range (TIR) derived from CGM (70∼180 mg/dl) shows good linear correlation with HbA1C and is expected to become a core indicator for short-term blood glucose assessment and the risk of diabetic complications [1]._._ In 2019, ADA guidelines were for the first time recommended in the International Consensus on TIR (glucose target time), suggesting a TIR target for type 2 diabetes mellitus (T2DM) patients with a range of 3.9 to 10.0 mmol/L.

It has been shown that a 5% increase in TIR was associated with significant clinical benefit in patients with T2DM [2]. However, the relationship between TIR and diabetic complications has not been fully studied, and whether TIR value resulting from the extensive fingertip glucose monitoring and non-GCM is equally meaningful remains to be investigated.

In this study, TIR values were calculated on the information-based management big data platform to further explore the relationship between TIR and HbA1c, as well as the risk of hypoglycemia. The relationship between TIR and diabetic microvascular complications, including diabetic retinopathy (DR), diabetic peripheral neuropathy (DPN), and diabetic nephropathy (DN) was also analyzed. We aim to provide a further theoretical basis for the clinical application of TIR and to explore a more suitable TIR control range for diabetic patients.

## 2. Materials and Methods

### 2.1. Data Sources

Big Data Sample Center of information Office of the Second Affiliated Hospital of Nanchang University provides data support. Information management Software was produced by DHC Software Co. Ltd.

### 2.2. Study Population

From August 2017 to June 2020, a total of 5,644 diabetic patients were hospitalized in the Endocrinology Department of the Second Affiliated Hospital of Nanchang University, and a total of 455,664 times of fingertip blood glucose monitoring were obtained. Patients who were diagnosed as nondiabetic or diabetic ketosis were excluded. A total of 3,408 patients who has more than 7 blood glucose points for three consecutive days after admission were selected as study subjects, and 71,568 blood glucose data were available. Relevant information was obtained based on the patient's ADM (unique medical code) information platform: gender, age, diabetes course, low density cholesterol, triglyceride, uric acid, glycosylated hemoglobin, urinary microalbumin, urinary albumin-to-creatinine ratio, and diabetic microvascular complications. The basic information for the patients is addressed in Table 1.

### 2.3. Diagnostic Criteria

Clinical diagnosis of diabetic nephropathy based on twice urinary microalbumin >30 mg/L or eGFR decreases, while excluding other CKD. Diagnostic criteria for diabetic peripheral neuropathy: if the patient has clinical symptoms (pain, numbness, paresthesia, etc.), any one of the 5 examinations (ankle reflex, acupuncture pain, vibration, pressure, and temperature) is abnormal according to the patient's diagnosis of neuropathy during or after diabetes. In the absence of clinical symptoms, 2 out of 5 abnormalities or abnormalities in electroneurogram examination can be diagnosed with DPN. The diagnosis of diabetic retinopathy is based on the results of dilated pupil or nondilated pupil fundus examination. TIR values were grouped by each 10 percentage points, and the correlation between TIR and HbA1c was analyzed.

### 2.4. Statistical Methods

Measurement data are represented as mean ± SD or median (Q1, Q3). Standard *t*-test was used for continuous variables with normal distributions for comparisons between groups. Kruskal–Wallis test was used for variance heterogeneity between groups. Chi-square test was used for comparison between count data sets. Single correlation analysis was performed using Pearson or Spearman method. All the *P* values were two sided, and *P* < 0.05 was considered statistically significant. Risk assessment was used to assess the relationship between TIR and diabetic microvascular complications. Statistical analysis was conducted using SPSS version 22.0. Statistical graphs were generated using GraphPad Prism 8.

## 3. Results

**Figure 1:**
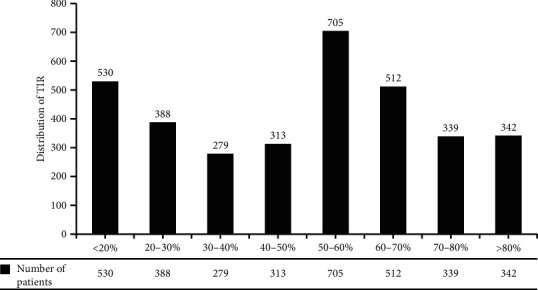
Distribution of TIR.

A total of 3,408 diabetes patients were included in the analysis, including 530 with TIR <20%, 388 with TIR of 20–30%, 279 with TIR of 30–40%, 313 with TIR of 40–50%, 705 with TIR of 50–60%, 512 with TIR of 60–70%, 339 with TIR of 70–80%, and 342 with TIR >80% (Figure 1).

There were 1,296 patients diagnosed with diabetic peripheral neuropathy (DPN), 2,077 patients diagnosed with diabetic retinopathy (DR), 836 patients diagnosed with diabetic nephropathy (DN), and 380 patients with DN who showed abnormal urinary albumin-to-creatinine ratio (UACR).

### 3.1. Correlation between TIR and HbA1C

With the decrease of TIR, HbA1C increased significantly, and the difference was statistically significant (*P* < 0.01, *R*^2^ = 0.458) (Table 2). The correlation coefficient of mean TIR with mean HbA1C was −0.626.

### 3.2. Correlation between Abnormal Urinary ACR and DN

**Figure 2:**
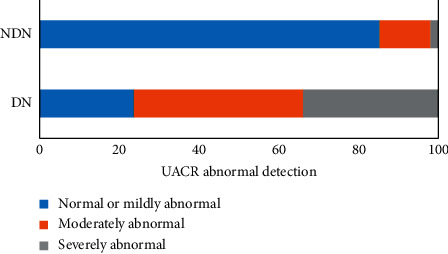
The correlation of DN with ACR.

Among 2572 nondiabetic nephropathy patients, 885 had valid ACR data. According to UACR, 732 patients (82.71%) had normal or mild abnormalities, 121 patients (13.67%) had moderate abnormalities, and 32 patients (3.62%) had severe abnormalities, with statistically significant differences (*χ*^2^ = 454.74, *P* < 0.01). Among 836 patients with diabetic nephropathy, 380 patients had valid ACR data, with an average ACR of 12.685 (4.99, 62.83). According to UACR, there were 90 patients (23.68%) with normal or mild abnormalities, 161 patients (42.37%) with moderate abnormalities, and 129 patients (33.95%) with severe abnormalities, with statistically significant differences (*χ*^2^ = 497.55, *P* < 0.01) (Figure 2).

### 3.3. Risk Assessment and Analysis of TIR and ACR Abnormal Occurrence

**Figure 3:**
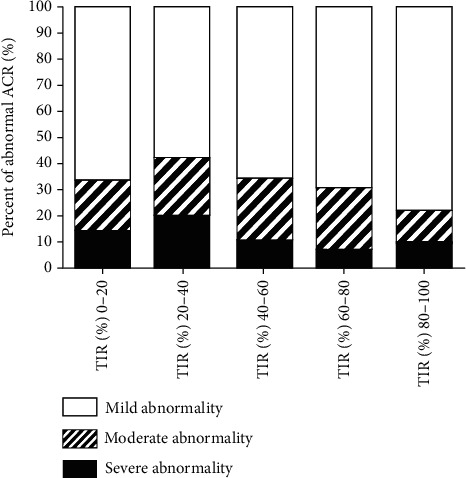
The correlation of TIR with ACR.

Risk assessment showed that TIR less than 40% was a risk factor for ACR abnormalities. According to the ACR value, the patients were divided into mild, moderate, and severe, and the TIR difference between the groups was statistically significant (*χ*^2^ = 497.55, *P* < 0.01) (Figure 3).

### 3.4. Risk Assessment and Analysis of TIR and DN

**Figure 4:**
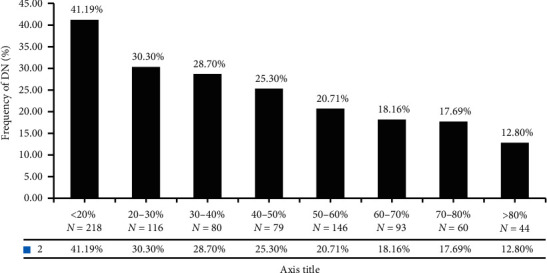
Distribution of DN with different TIR.

There were 836 patients diagnosed DN. TIR value between DN and non-DN was significant (*T* = 2.250, *P* < 0.05). Risk assessment showed the lower the TIR was, the higher the risk of DN was. TIR less than 40% was a risk factor for DN (OR = 1.249, 95% CI: 0.930–1.679) (Figure 4).

### 3.5. Risk Assessment and Analysis of TIR and DPN

**Figure 5:**
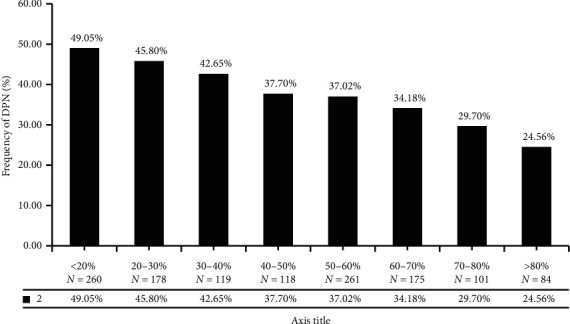
Distribution of DPN with different TIR.

There were 1,296 patients diagnosed DPN. TIR value between DPN and non-DPN was significantly different (*T* = 3.844, *P* < 0.01). Risk assessment shows that the lower the TIR was, the higher the risk of DPN was. TIR value less than 70% is a risk factor for DPN (OR = 1.030, 95% CI: 0.769–1.379). TIR less than 20% was twice the risk of DPN than greater 80% (Figure 5).

### 3.6. Risk Assessment and Analysis of TIR and DR

**Figure 6:**
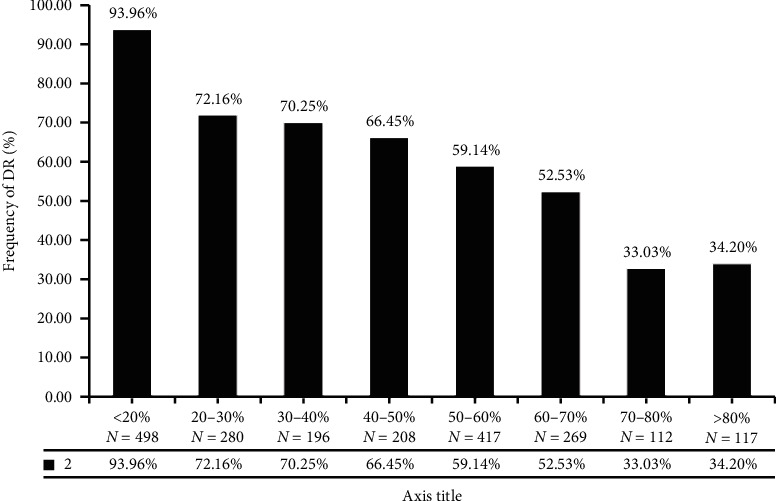
Distribution of DR with different TIR.

There were 2,077 patients diagnosed DR, and the TIR value between DR and nondiabetic retinopathy was significantly different (*T* = 3.006, *P* < 0.05). Risk assessment shows the lower the TIR value was, the higher the risk of DR was (*χ*^2^ = 509.739 *P* < 0.01). TIR value less than 50% is a risk factor for DPN (OR = 1.092, 95% CI: 0.898–1.264). TIR less than 20% was twice the risk of DPN than greater 80% (Figure 6).

### 3.7. Correlation between TIR Quartile and Diabetic Microangiopathy

**Figure 7:**
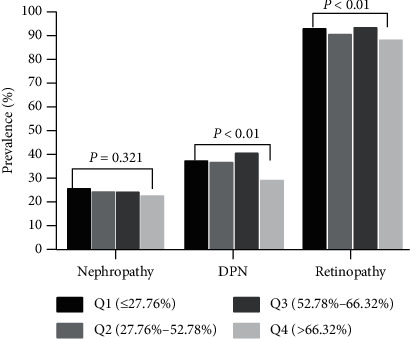
Relationship between TIR quartile and microangiopathy.

The effect of TIR on diabetic microvascular lesions was analyzed by TIR quartile grouping. The results showed that the lower the TIR value was, the higher the prevalence of diabetic peripheral neuropathy and retinopathy was, and the difference was statistically significant (*χ*^2^ = 25.596, *P* < 0.001; *χ*^2^ = 17.779, *P* < 0.001). However, there was no statistically significant difference in the analysis of quartile nephropathy (*χ*^2^ = 3.502, *P* − 0.321) (Figure 7).

## 4. Discussion

As a major chronic noncommunicable disease threatening human health, diabetes has become an inevitable public health challenge worldwide due to its high incidence, high disability rate, and high mortality rate. How to predict, prevent and, reduce the occurrence and development of diabetes complications more accurately has emerging and strong demand in diabetes management. In particular, appropriate blood glucose management is known to reduce the occurrence and development of complications. In contrast, persistent hyperglycemia or glucose fluctuation is closely related to the occurrence and development of diabetic complications. Therefore, keeping blood glucose within a healthy range and limiting blood glucose fluctuation in the early stage of the disease can effectively reduce the risk of T2DM-related microvascular and macrovascular complications [3].

Glycosylated hemoglobin (HbA1C) that reflects the average blood glucose level in the past 2 to 3 months has long been used as an important basis for long-term blood glucose control and a gold indicator predicting long-term complications of diabetes. Improved HbA1c dictates lower risk of microvascular and macrovascular disease [4]. However, more and more evidence show that HbA1c still has certain limitations. For instance, HbA1c cannot provide information related to daily hypoglycemia or hyperglycemia or short-term blood glucose fluctuations. The level of HbA1c is affected by multiple factors such as the amount of hemoglobin and the life span of hemoglobin in bloodstream of the patients. Consequently, it may not accurately reflect the blood glucose control of patients with anemia, hemoglobin disease, iron deficiency anemia, pregnancy, and at other status. For individuals, information from patients with elevated HbA1C is not specific enough for clinicians *w* to adjust treatment regimens [5, 6].

Although CGM can retrospectively provide 24-hour blood glucose monitoring data, high cost of equipment and the accuracy requirement of frequent fingertip blood glucose have become significant barriers to the widespread clinical use of CGM. In recent years, the concept beyond glycosylated hemoglobin has been proposed, and the target time in range (TIR) blood glucose value (generally defined as 3.9∼10.0 mmol/L) generated by CGM has become hotspot in research for the clinical efficacy and risk assessment of diabetic complications [7].

Vigersky and Mc Mahon have analyzed 1,137 patients with type 1 and type 2 diabetes in 18 literature studies and investigated the correlation between TIR and HbA1C, which showed that TIR (3.9–10.0 mmol/L) was highly correlated with HbA1C [1]. Consistently, DR in type 2 diabetes has been shown to be associated with TIR derived from CGM in another cross-sectional study. This evidence suggests that TIR is a very promising core indicator for clinical glycemic assessment, and the risk of diabetic complications [8], including complications such as urinary microalbumin-to-urinary creatinine ratio (ACR), diabetic peripheral neuropathy, cardiovascular disease, and other diabetic complications has not been publicly reported.

In this study, information management platform has provided big data resource of 3,408 hospitalized patients with T2DM. TIR value was calculated by 7-point blood glucose monitoring for 3 consecutive days, and the relationship between TIR and HbA1C and the risk of microvascular complications was also analyzed. We found that lower TIR indicates higher risk of microvascular complications including DPN, DR, and DN, as well as higher risk of ACR abnormalities.

It is reasonable to speculate that the correlation between TIR measured by the seven-point test and complications would also be consistent with the CGM-derived TIR, which has been demonstrated by three studies suggesting similar TIR results comparing CGM and fingertip glucose measurements. In a study conducted by the Diabetes Research in Children Network (DirecNet), the mean TIR of the total 161 subjects was 49%, measured using CGM and 50% measured with eight-point testing (an overnight measurement added to the seven-point profile) [9]. In another study combining data from six inpatient studies, the mean TIR was 60% with both CGM and with paired blood glucose measurements made with an YSI analyzer or by a central laboratory [10]. Beck et al. retrospectively analyzed the relationship between target blood glucose TIR (calculated from 7-point self-monitoring blood glucose (SMBG)) and HbA1C in a DCCT study, and the results showed that a 10% increase in TIR corresponded to an average decrease of 0.9% in HbA1C level in patients with type 1 diabetes [11]. In this study, the mean TIR value was 49.65%, close to the mean TIR value of fingertip blood glucose monitoring in Beck's study, but it was higher than the average value of TIR obtained by CGM in general. This may be due to the fact that the subjects in our study were hospitalized patients and the intervention was more frequent and active. In addition, TIR in our data has a certain negatively correlation with HbA1C, suggesting that TIR value could be a potential indicator of HbA1C.

Compared with using CGM data, using seven-point test data has several limitations. The abilities to assess the blood glucose change with individuals and between individuals were compromised. In addition, 7 points of data may not include information from overnight periods, which may lead to underestimation of TIR; however, this is not likely to impact the association of TIR with diabetic complications [12]. In this study, only observational studies were performed, without intervention analysis before and after treatment. Further studies on the impact of different treatments on TIR outcomes in diabetic patients are expected.

## 5. Summary

Based on the data above, we conclude that the TIR values derived from the seven-point test produce similar results as the values generated from CGM and TIR are closely associated with the risk of diabetic microvascular complications. In summary, TIR could be considered as a promising indicator of short-term glycemic control and a predictor for the risks of long-term diabetic complications [13].

## Figures and Tables

**Table 1:** The basic information for the patients.

Complications		Age (year)	Course (year)	TIR (%)	HbA1C (%)	LDL-C (mmol/L)	TG (*µ*mol/L)	Uric acid (mmol/L)	Cr(mmol/L)
*DN*	Diagnosed	66.07 ± 11.97	10.0 (4.0,15.0)	41.94 ± 22.98	9.25 ± 2.62	3.53 ± 1.36	1.45 (1.04, 2.09)	398.29 ± 150.79	197.63 ± 75.79
	Nondiagnosed	57.68 ± 13.20	6 (1.00, 10.00)	52.15 ± 22.94	8.82 ± 2.44	3.55 ± 1.32	1.38 (0.97, 2.18)	337.20 ± 110.49	109.26 ± 39.71
	*F*/*Z*	11.92	−9.73	6.94	10.10	3.77	−1.28	27.82	57.68
	*P*	0.001	<0.01	<0.01	<0.01	0.052	0.202	<0.01	<0.001

*DPN*	Diagnosed	62.14 ± 12.59	8.50 (2.00, 13.00)	43.15 ± 22.91	9.20 ± 2.55	3.51 ± 1.26	1.33 (0.97, 1.94)	368.72 ± 133.26	169.63 ± 35.47
	Nondiagnosed	58.27 ± 13.67	6.00 (2.00, 11.00)	52.40 ± 23.21	8.76 ± 2.45	3.57 ± 1.38	1.45 (0.98, 2.33)	351.87 ± 126.16	128.56 ± 58.51
	*F*/*Z*	10.30	−4.95	2.15	−2.43	0.02	−3.01	0.33	−0.47
	*P*	0.001	<0.01	<0.05	<0.05	0.881	0.003	0.566	0.568

*DR*	Diagnosed	63.05 ± 13.10	8.00 (2.00, 13.00)	42.82 ± 22.58	9.29 ± 2.51	3.50 ± 1.21	1.35 (0.97, 2.02)	367.16 ± 132.91	173.65 ± 54.27
	Nondiagnosed	54.45 ± 12.12	6.00 (2.00, 10.00)	60.57 ± 20.24	8.34 ± 2.36	3.63 ± 1.49	1.48 (1.02, 2.45)	341.29 ± 119.84	121.78 ± 43.16
	*F*/*Z*	7.99	−3.70	64.51	11.35	2.20	−3.10	1.27	7.65
	*P*	0.005	<0.01	<0.01	<0.01	0.138	<0.05	0.260	<0.05

**Table 2:** HbA1c corresponding to every 10% TIR.

TIR (%)	HbA1C (%)
<10	13.76
10–20	11.33
20–30	10.19
30–40	9.45
40–50	9.08
50–60	8.61
60–70	8.09
70–80	7.47
80–90	6.60
90–100	5.62

## Data Availability

Big Data Sample Center of Information Office of the Second Affiliated Hospital of Nanchang University provided data support. Information management Software was produced by DHC Software Co. Ltd. The raw data can available upon request to Xia Sheng via email (156934289@qq.com).
